# Statistical stopping criteria for automated screening in systematic reviews

**DOI:** 10.1186/s13643-020-01521-4

**Published:** 2020-11-28

**Authors:** Max W Callaghan, Finn Müller-Hansen

**Affiliations:** 1 Mercator Research Institute on Global Commons and Climate Change, EUREF Campus 19, Torgauer Straße 12-15, Berlin, 10829 Germany; 2 Priestley International Centre for Climate, University of Leeds, Leeds, LS2 9JT UK; 3 Potsdam Institute for Climate Impact Research (PIK), Member of the Leibniz Association, P.O. Box 60 12 03, Potsdam, 14412 Germany

**Keywords:** Systematic review, Machine learning, Active learning, Stopping criteria

## Abstract

Active learning for systematic review screening promises to reduce the human effort required to identify relevant documents for a systematic review. Machines and humans work together, with humans providing training data, and the machine optimising the documents that the humans screen. This enables the identification of all relevant documents after viewing only a fraction of the total documents. However, current approaches lack robust stopping criteria, so that reviewers do not know when they have seen all or a certain proportion of relevant documents. This means that such systems are hard to implement in live reviews. This paper introduces a workflow with flexible statistical stopping criteria, which offer real work reductions on the basis of rejecting a hypothesis of having missed a given recall target with a given level of confidence. The stopping criteria are shown on test datasets to achieve a reliable level of recall, while still providing work reductions of on average 17%. Other methods proposed previously are shown to provide inconsistent recall and work reductions across datasets.

## Background

Evidence synthesis technology is a rapidly emerging field that promises to change the practice of evidence synthesis work [1]. Interventions have been proposed at various points in order to reduce the human effort required to produce systematic reviews and other forms of evidence synthesis. A major strand of the literature works on screening: the identification of relevant documents in a set of documents whose relevance is uncertain [2]. This is a time-consuming and repetitive task, and in a research environment with constrained resources and increasing amounts of literature, this may limit the scope of the evidence synthesis projects undertaken. Several papers have developed active learning (AL) approaches [3–7] to reduce the time required to screen documents. This paper sets out how current approaches are unreliable in practice, and outlines and evaluates modifications that would make AL systems ready for live reviews.

Active learning is an iterative process where documents screened by humans are used to train a machine learning model to predict the relevance of unseen papers [8]. The algorithm chooses which studies will next be screened by humans, often those which are likely to be relevant or about which the model is uncertain, in order to generate more labels to feed back to the machine. By prioritising those studies most likely to be relevant, a human reviewer most often identifies all relevant studies—or a given proportion of relevant studies (described by recall: the number of relevant studies identified divided by the total number of relevant studies)—before having seen all the documents in the corpus. The proportion of documents not yet seen by the human when they reach the given recall threshold is referred to as the work saved. This represents the proportion of documents that they do not have to screen, which they would have had to without machine learning.

Machine learning applications are often evaluated using sets of documents from already completed systematic reviews for which inclusion or exclusion labels already exist. As all human labels are known a priori, it is possible to simulate the screening process, recording when a given recall target has been achieved. In live review settings, however, recall remains unknown until all documents have been screened. In order for work to really be saved, reviewers have to stop screening while uncertain about recall. This is particularly problematic in systematic reviews because low recall increases the risk of bias [9]. The lack of appropriate stopping criteria has therefore been identified as a research gap [10, 11], although some approaches have been suggested. These have most commonly fallen into the following categories:

- *Sampling criteria*: Reviewers estimate the number of relevant documents by taking a random sample at the start of the process. They stop when this number, or a given proportion of it, has been reached [12].
- *Heuristics*: Reviewers stop when a given number of irrelevant articles are seen in a row [6, 7].
- *Pragmatic criteria:* Reviewers stop when they run out of time [3].
- *Novel automatic stopping criteria:* Recent papers have proposed more complicated novel systems for automatically deciding when to stop screening [13–15].

We review the first three classes of these methods in the following section and discuss their theoretical limitations. They are then tested on several previous systematic review datasets. We demonstrate theoretically and with our experimental results that these three classes of methods cannot deliver consistent levels of work savings or recall—particularly across different domains, or datasets with different properties [2]. We also discuss the limitations of novel automatic stopping criteria, which have all demonstrated promising results, but do not achieve a given level of recall in a reliable or reportable way. Without the reliable or reportable achievement of a desired level of recall, deployment of AL systems in live reviews remains challenging.

This study proposes a system for estimating the recall based on random sampling of remaining documents. We use a simple statistical method to iteratively test a null hypothesis that the recall achieved is less than a given target recall. If the hypothesis can be rejected, we conclude that the recall target has been achieved with a given confidence level and screening can be stopped. This allows AL users to predefine a target in terms of uncertainty and recall, so that they can make transparent, easily communicable statements like “We reject the null hypothesis that we achieve a recall of less than 95% with a significance level of 5%”.

In the remainder of the paper, we first discuss in detail the shortcomings of existing stopping criteria. Then, we introduce our new criteria based on a hypergeometric test. We evaluate our stopping criteria and compare their performance with heuristic- and sampling-based criteria on real-world systematic review datasets on which AL systems have previously been tested [13, 16–18].

## Methods review

We start by explaining the sampling- and heuristic-based stopping criteria and discussing their methodological limitations.

### Sampling-based stopping criteria

The stopping criterion suggested by Shemilt et al. [12] involves establishing the Baseline Inclusion Rate (BIR), by taking a random sample at the beginning of screening. The BIR is used to estimate the number of relevant documents in the whole dataset. Reviewers continue to screen until this number, or a proportion of it corresponding to the desired level of recall, is reached.

However, the estimation of the BIR fails to correctly take into account sampling uncertainty.^1^ This uncertainty is crucial, as errors can have severe consequences. Let us assume that users will stop screening when they have identified 95% of the relevant number of documents. If the estimated number of relevant documents is more than the true number of relevant documents divided by 0.95, then the users will never see 95% of the estimated number. This means that they will keep screening until they have seen all documents, and no work savings will be achieved. Conversely, if the number of relevant documents is underestimated by even a single unit, then the recall achieved will be lower than the target.

**Fig. 1:**
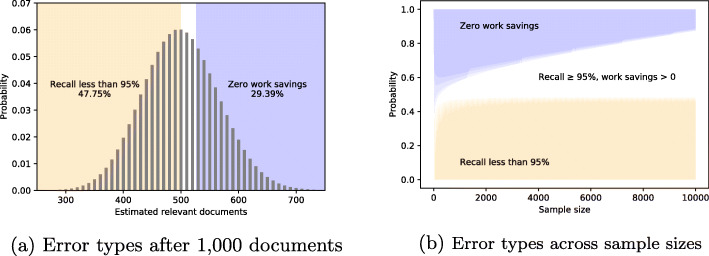
Distribution of under- or overestimation errors using the BIR sampling method in a dataset of 20,000 documents of which 500 are relevant. **a** The probability distribution of the estimated number of relevant documents after a sample of 1000 documents. **b** The probability of each type of error according to the sample size

The number of relevant documents drawn without replacement from a finite sample of documents follows the hypergeometric distribution. Figure 1a shows the distribution of the predicted number of documents after drawing 1000 documents from a total of 20,000 documents, where 500 documents (2.5%) are relevant. The left shaded portion of the graph shows all the cases where the recall will be less than 95%. This occurs 48% of the time. The right shaded portion of the graph shows the cases where the number of relevant documents is overestimated so much that no work savings could be made to achieve a target recall of 95%. This occurs 29% of the time. In only 23% of cases can work savings be achieved while still achieving a recall of at least 95%.

Figure 1b shows the probability distribution of these errors according to the sample size. Even with very large samples, both types of error remain frequent. This shows how baseline estimation inevitably offers poor reliability, either in terms of recall or in work saved.

#### Heuristic stopping criteria

Some studies give the example of heuristic stopping criteria based on drawing a given number of irrelevant articles in a row [6, 7]. We take this as a proxy for estimating that the proportion of remaining documents that are relevant in the unseen documents is low, as the probability of observing 0 relevant documents in a given sample (analogous to a set of consecutive irrelevant results) is a decreasing function of the number of relevant documents in the population. We find this a promising intuition, but argue that (1) it ignores uncertainty, as discussed in relation to the previous method; (2) it lacks a formal description that would help to find a suitable threshold for the criterion; and (3) it misunderstands the significance of a low proportion of relevant documents in estimating the recall.

**Fig. 2:**
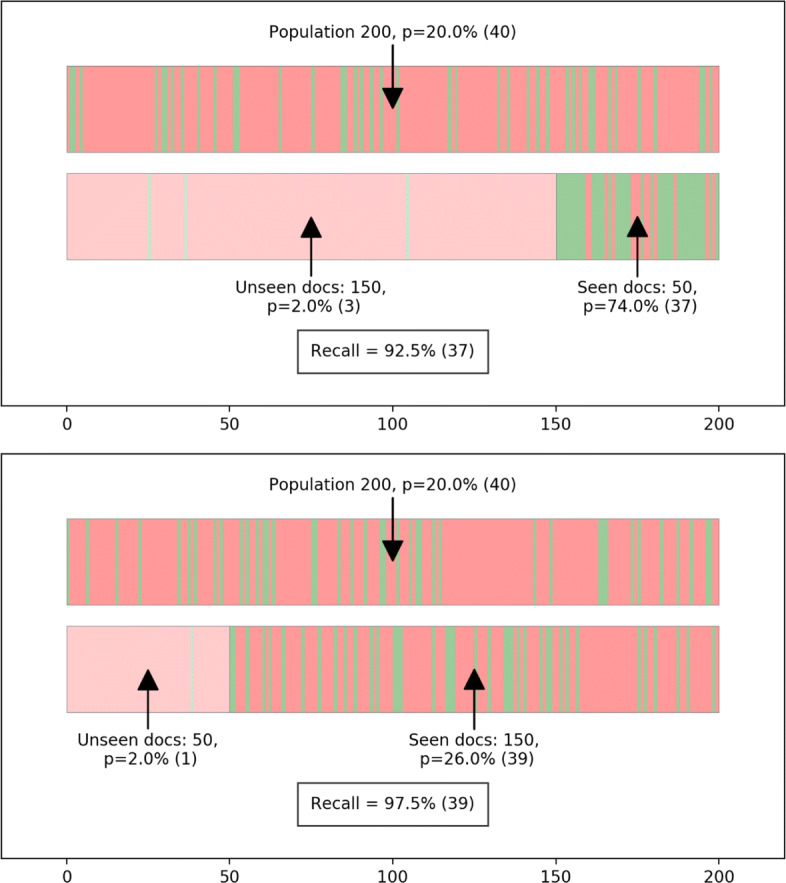
Similar low proportions of relevant documents in unseen documents with different consequences for recall. The top bar shows a random distribution of relevant documents (green) and irrelevant documents (red) at a given proportion of relevance. The bottom bar shows distributions of relevant and irrelevant documents in hypothetical sets of seen (right) and unseen (left—transparent) documents

Figure 2 illustrates this third point. We show two scenarios with identical low proportions of relevant documents observed in the unseen documents. In the top part of the figure, machine learning (ML) has performed well, and 74% of the screened documents were relevant. In the bottom part of the figure, ML has performed less well, and only 26% of the screened documents were relevant. In both cases, only 2% of unseen documents are relevant, but 2% of a larger number means more relevant documents are missed. Recall is not simply a function of the proportion of unseen documents that are relevant, but also of the number of unseen documents. This also means that where ML has performed well (as in the top figure), a low proportion of relevant documents in those not yet checked is indicative of lower recall than where ML has performed less well. Likewise, where the proportion of relevant documents in the whole corpus is low, a similarly low proportion of relevant documents is likely to be observed, even when true recall is low. This shows us that even a perfect estimator of the proportion of unseen documents that are relevant is insufficient on its own to provide sufficient information about when to stop screening. To estimate recall reliably, it is necessary to take into account the total number of unseen relevant documents (or their proportion times the number of unseen documents).

#### Pragmatic stopping criteria

Wallace et al. [4] develop a “simple, operational stopping criterion”: stopping after half the documents have been screened. Although the criterion worked in their experiment, it is unclear how this could be generalised, and its development depended on knowledge of the true relevance values. Jonnalagadda and Petitti [6] note that “the reviewer can elect to end the process of classifying documents at any point, recognizing that stopping before reviewing all documents involves a trade-off of lower recall for reduced workload”, although clearly the reviewer lacks information about probable recall.

#### Novel automatic stopping criteria

Two examples come from the information retrieval literature. Di Nunzio [14] presents a novel automatic stopping criterion based on BM25, although recall reported is “often between 0.92 and 0.94 and consistently over 0.7”. Yu and Menzies [13] also present a stopping criterion based on BM25 which allows the user to target a specific level of recall. However, reviewers are not given the opportunity to specify a confidence level, and for two of the four datasets in which they tested their criteria, the median achieved recall at a stopping criteria targeting 95% recall was below 95%. In each case, the reliability of the estimate is dependent on the performance of the model.

Finally, Howard et al. [15] present a method to estimate recall based on the number of irrelevant documents *D* observed in a list of documents since the *δ*th previous relevant document. They reason that this should follow the negative binomial distribution based on the proportion of remaining relevant documents *p*, and use this information to estimate $\hat {p}$, and with this, the total number of relevant articles and the estimated recall.

However, their method does not quantify uncertainty, but can only claim that the method “*tends* to result in a conservative estimate of recall” (emphasis ours). This is not guaranteed by the criterion itself but rather a finding of the simulation with example datasets. Further, the authors do not give sufficient information to reproduce their results, providing neither code (they describe their own proprietary software) nor an equation for $\hat {p}$. Additionally, the criterion requires a tuning parameter *δ*, which users may have insufficient information to set optimally. Lastly, because screening is a form of sampling without replacement, the negative hypergeometric distribution should be preferred to the negative binomial, even though the latter can be a good approximation for cases with large numbers of documents.

These last examples are promising developments, but they all fail to take into account the needs of live systematic reviews, where the reliability of and ease of communication about recall are paramount, and the results must be independent of model performance. In the following, we explain our own method, which provides clearly communicable estimates of recall, and which manages uncertainty in a way robust to model performance.

## Methods

### A statistical stopping criterion for active learning

In our screening setup, we start off with *N*_*tot*_ documents that are potentially relevant. *ρ*_*tot*_ of these documents are actually relevant, but we do not know this value a priori. As we screen relevant documents, we include them, so *ρ*_*seen*_ represents the number of relevant documents screened, and recall *τ* is given by:

1 $$ \tau = \frac{\rho_{seen}}{\rho_{tot}}   $$

**Fig. 3:**
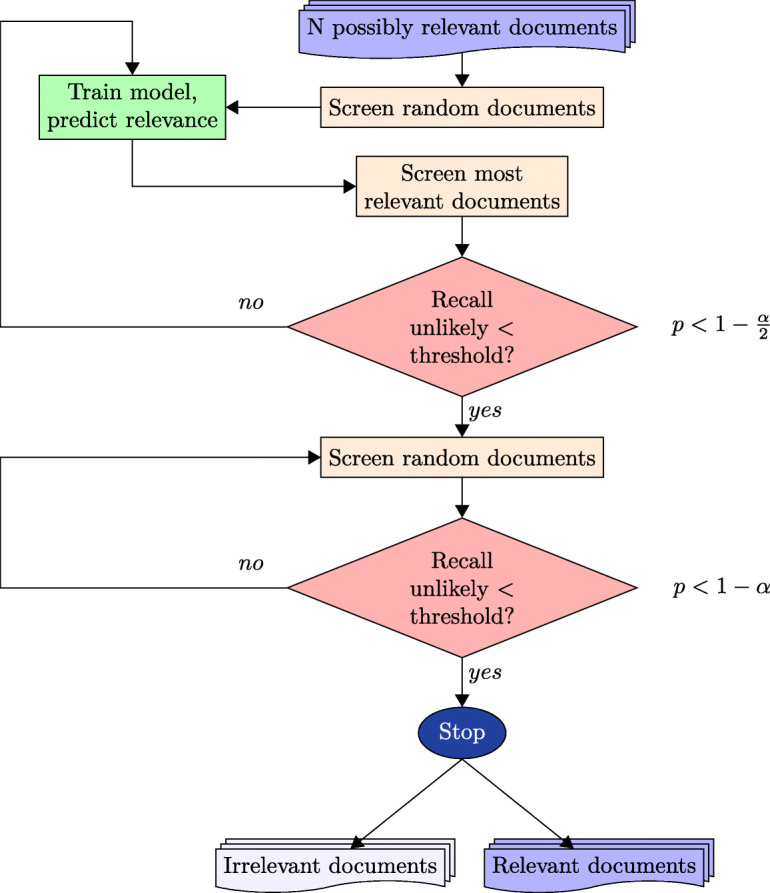
A workflow for active learning in screening with a statistical stopping criterion

We set a target recall *τ*_*tar*_ and a confidence level *α*. We want to keep screening until *τ*≥*τ*_*tar*_, and devise a hypothesis test to estimate whether this is the case with a given level of confidence (Fig. 3). We do this based on interrupting the active-learning process and drawing a random sample from the remaining unseen documents. We first describe this test, before showing how a variation on the test can be used to decide when to begin drawing a random sample.

#### Random sampling

At the start of the sample, *N*_*AL*_ is the number of documents seen during the active learning process, and *N* is the number of documents remaining, so that:

2 $$ N = N_{tot} - N_{AL}  $$

We refer to the number of relevant documents seen during active learning as *ρ*_*AL*_, and the number of remaining relevant documents as *K*. We do not know the value of *K* but know that it is given by the total number of relevant documents minus the number of relevant documents seen during active learning.

3 $$ K = \rho_{tot} - \rho_{AL}   $$

We now take random draws from the remaining *N* documents, and denote the number of documents drawn with *n* and the number of relevant documents drawn with *k*. The number of relevant documents seen is updated by adding the number of relevant documents seen since sampling began to the number of relevant documents seen during active learning.

4 $$ \rho_{seen} = \rho_{AL} + k   $$

We proceed to form a null hypothesis that the true value of recall is less than our target recall:

5 $$ H_{0} : \tau < \tau_{tar}   $$

Accordingly, the alternative hypothesis is that recall is equal to or greater than our target:

6 $$ H_{1} : \tau \geq \tau_{tar}  $$

Because we are sampling without replacement, we can use the hypergeometric distribution to find out the probability of observing *k* relevant documents in a sample of *n* documents from a population of *N* documents of which *K* are relevant. We know that *k* is distributed hypergeometrically:

7 $$ k \sim \text{Hypergeometric}(N, K, n)  $$

We introduce a hypothetical value for *K*, which we call *K*_*tar*_. This represents the minimum number of relevant documents remaining at the start of sampling compatible with our null hypothesis that recall is below our target.

8 $$ K_{tar} = \lfloor \frac{\rho_{seen}}{\tau_{tar}}-\rho_{AL}+1 \rfloor  $$

This equation is derived by combining Eqs. 1 and 4. Because *k* can only take integer values, *K*_*tar*_ is the smallest integer that satisfies the inequality in Eq. 5. With *K*_*tar*_, we can reformulate our null hypothesis: the true number of relevant documents in the sample is greater than or equal to our hypothetical value.

9 $$ H_{0} : K \geq K_{tar}  $$

We test this by calculating the probability of observing *k* or fewer relevant documents from the hypergeometric distribution given by *K*_*tar*_, using the cumulative probability mass function.

10 $$ p = P(X \leq k), \text{where}\ X \sim \text{Hypergeometric}(N,K_{tar},n)   $$

Because the cumulative probability mass function *P*(*X*≤*k*) is decreasing with increasing *K*, this gives the maximum probability of observing *k* for all values of *K* compatible with our null hypothesis. Similar arguments have been made to derive confidence intervals for estimating the parameter *K* in the hypergeometric distribution function [19, 20], and the derivation of an equivalent criterion could use the upper limit of such a confidence interval of an estimated *K* from the observation of *k*.

We can reject our null hypothesis and stop screening if the maximum probability of obtaining our observed results given our null hypothesis *p* is below 1−*α*.^2^ To further investigate the accuracy of the test, we perform an experiment drawing 1 million random samples in 6 scenarios with different characteristics. We vary the value of *ρ*_*AL*_ to simulate starting random sampling with different levels of recall achieved.

**Fig. 4:**
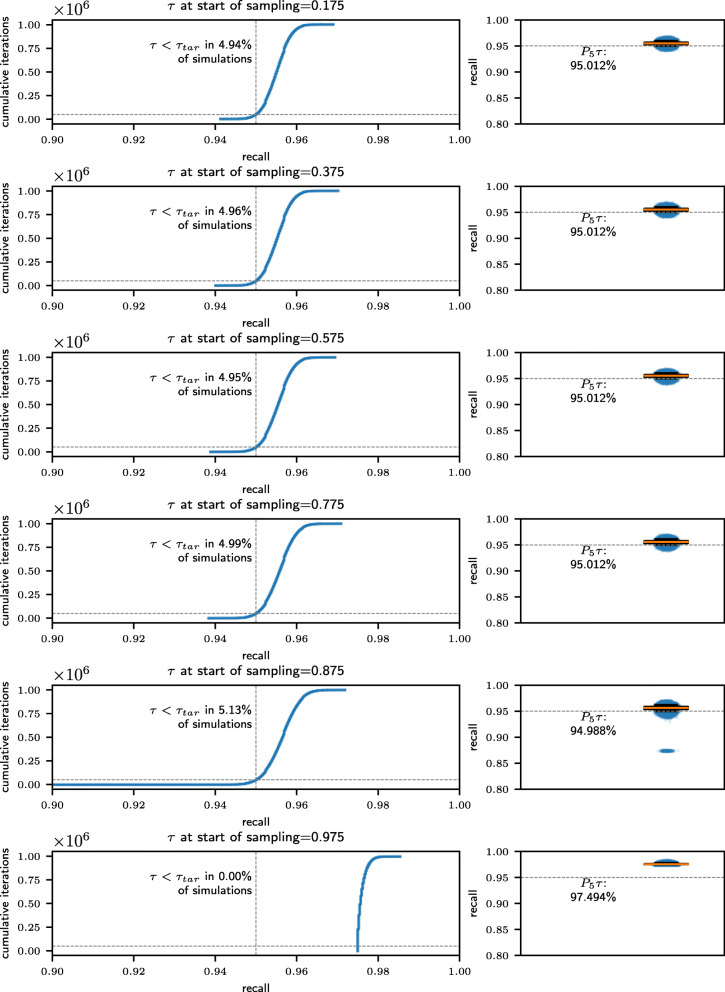
The distribution of achieved recall values given our random sampling stopping criterion for 6 scenarios with different recall values at the start of sampling

Figure 4 shows that in each case, as long as recall is lower than the target recall when sampling begins, the percentage of trials in which the criterion is trigerred to early is within two tenths of a percentage point of 5% and the 5th percentile of achieved recall values is within two tenths of a percentage point of the target recall 95%.

#### Ranked quasi-sampling

We now proceed to describe a special case of the method described above which we (1) use as a heuristic in order to decide when to begin random sampling and (2) test as an independent stopping criterion. The method works by treating batches of previously screened documents as if they were random samples.

We calculate *p* as above for subsets of the already screened documents. Concretely, we use subsets of documents *A*_*i*_ by looking back to the last *i* documents, $\phantom {\dot {i}\!}A_{i} = \{d_{N_{seen} - 1},..., d_{N_{seen} - i}\}$, where the documents *d* are indexed in the order in which they have been screened. For a specific *i*, this corresponds to random sampling beginning after seeing *i* documents in the section above. Thus, we set *N*_*AL*_ to *i*, *n* to *N*_*seen*_−*i*,*ρ*_*AL*_ to the number of relevant documents seen when *i* documents had been seen, and *k* to the number of relevant documents seen since *i* documents had been seen, and calculate *p* according to Eq. 10. We compute *p* for all sets *A*_*i*_ with $i \in {N_{seen}-1 \dots 1}$. This gives us a vector *p*, representing the values of *p* which would have been estimated at each point at which we could have stopped active learning and began random sampling. The point at which the *p* value for our null hypothesis is lowest is given by *p*_*min*_. With the vectorised implementation included in our accompanying code, these calculations are completed in less than the time it would take a human to code the next document.

First, we use this method as a useful heuristic for deciding when to stop active learning, and switch to random sampling. For this, we choose a higher threshold for the likelihood, $p_{min} < 1-\frac {\alpha }{2}$. Second, we use the same ranked quasi-sampling as an independent stopping criterion, by continuing screening with active learning until *p*_*min*_<1−*α*. We present the results of this second procedure separately below.

Given that the documents seen during active learning are ranked according to predicted relevance, they do not in fact represent a random sample. This means that the test is unlikely to be accurate. It would be reasonable to assume that the proportion of relevant documents in each ranked quasi-sample is as high if not higher than the proportion of relevant documents in the unseen documents. This assumption would make this estimator conservative. As such, it works in a similar way to the criterion proposed by Howard et al. [15], although it makes use of more information and provides hypothesis testing rather than just a point estimate of recall.

### Evaluation

We evaluate each of the criteria discussed on real-world test data, operationalising the heuristic stopping criteria with 50, 100, and 200 consecutive irrelevant records. We run 100 iterations on each dataset and record the following measures:

- *Actual Recall*: The recall when the stopping criteria were met.
- *WS-SC*: Work saved when the stopping criteria were met.
- *Additional Burden*: The work saved when the criterion was triggered subtracted from the work saved when the recall target was actually achieved.

For simplicity, we use a basic SVM model [21, 22], with 1–2 word n-grams taken from the document abstracts used as input data. We start with random samples of 200 documents (we do not employ Shemilt et al.’s methods for identifying the “optimal” sample size, as we showed these in the “Methods review” section to be unhelpful). Subsequently, we “screen”, that is, we reveal the labels of, batches of the 20 documents with the highest predicted relevance scores, retraining the model after each batch. Theoretically, using smaller batch sizes could mean that the recall target is achieved more quickly, but this is a trade-off between computational time spent training and the speed at which the algorithm can “learn”. However, this is a modelling choice which may affect work saved, but not recall. Each criterion is evaluated after each document is “screened”. For our criteria, we set the target recall value to 95% and the confidence level to 95%.

The systematic review datasets used for testing are described in Table 1. We use the seminal collection of systematic reviews used to develop machine learning applications for document screening by Cohen and co-authors in 2006 [16], along with the widely used Proton Beam [17] and COPD [18] datasets, and computer science datasets used to test FASTREAD [13]. Testing on datasets with different properties and from different domains is key to establishing criteria appropriate for general use. Choosing as broad as possible data also prevents us from being able to “tune” our machine learning approach in ways that may work well for specific datasets but not generalise well. Work savings, even maximum work savings, are therefore below the state of the art recorded for each of these datasets. In this way, we can show how well the criteria perform even when the model performs badly.

**Table 1:** Dataset properties

	Dataset	Data source	N documents	N relevant documents	Proportion relevant documents
0	UrinaryIncontinence	cohen	284	68	0.24
1	Antihistamines	cohen	287	90	0.31
2	Estrogens	cohen	349	79	0.23
3	NSAIDS	cohen	358	83	0.23
4	OralHypoglycemics	cohen	475	135	0.28
5	Triptans	cohen	594	205	0.35
6	ADHD	cohen	803	83	0.10
7	AtypicalAntipsychotics	cohen	1030	333	0.32
8	CalciumChannelBlockers	cohen	1103	257	0.23
9	ProtonPumpInhibitors	cohen	1210	227	0.19
10	SkeletalMuscleRelaxants	cohen	1348	30	0.02
11	COPD	copd_pb	1443	179	0.12
12	Kitchenham	fastread	1700	45	0.03
13	Opiods	cohen	1769	43	0.02
14	BetaBlockers	cohen	1872	270	0.14
15	ACEInhibitors	cohen	2234	168	0.08
16	Statins	cohen	2743	152	0.06
17	ProtonBeam	copd_pb	4108	240	0.06
18	Radjenovic	fastread	5999	47	0.01
19	Wahono	fastread	7002	62	0.01
20	Hall	fastread	8911	104	0.01

All computational steps required to reproduce this analysis are documented online at https://github.com/mcallaghan/rapid-screening.

## Results

**Fig. 5:**
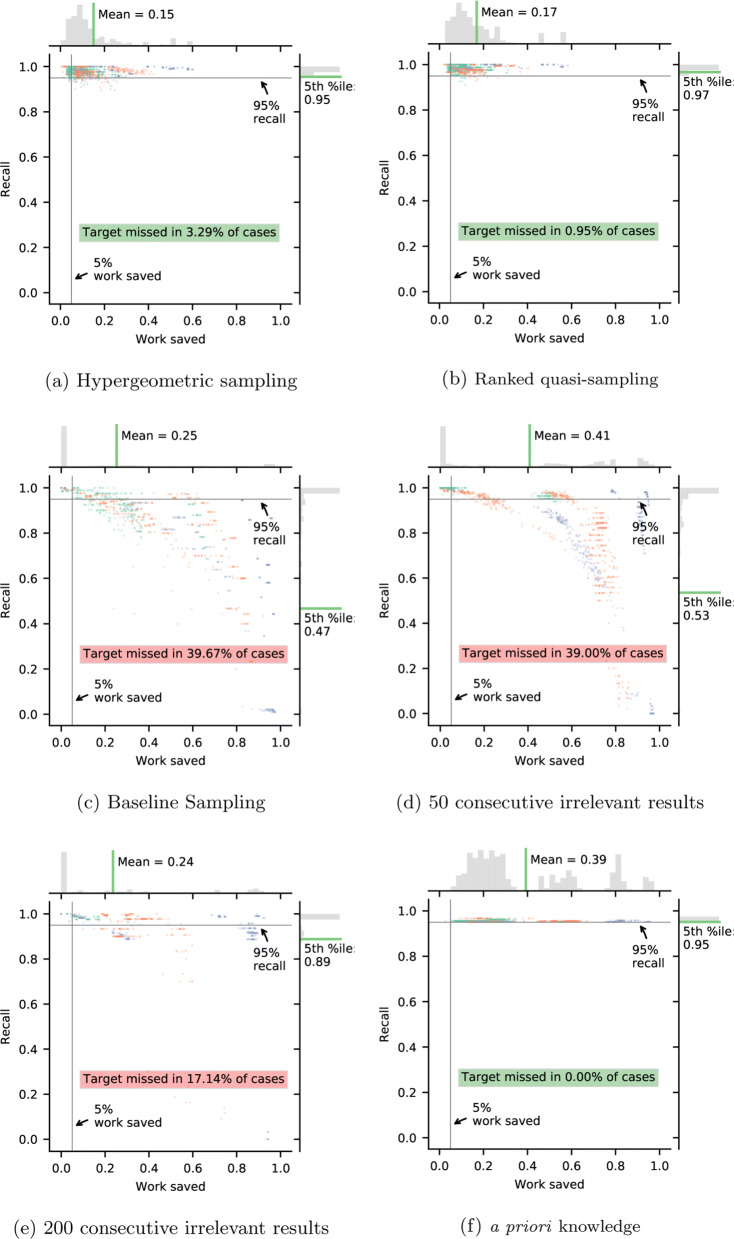
Distribution of recall and work saved after each stopping criteria. Green dots show results for datasets with less than 1000 documents, orange dots show datasets with 1000–2000 documents, and blue dots show datasets with more than 2000 documents

Figure 5 shows the actual recall and work savings achieved when each stopping criterion has been satisfied. For comparison, we also include the results that would have been achieved with a priori knowledge of the data, that is, the work saved when the 95% recall target was actually reached. In a live systematic review, reviewers would never know when this had been reached, but these are the work savings most often reported in machine learning for systematic review screening studies.

Both the random sampling and the ranked sampling criteria achieve the target threshold of 95% in more than 95% of cases. That this is greater than 95% is accounted for by the fact that random sampling sometimes begins after the target recall has been achieved, in which case the null hypothesis would be a priori impossible. The ranked quasi-sampling criterion outperforms the random sampling criterion with respect to both recall and work savings, saving a mean of 17% of the work compared to 15%, and missing the target in only 0.95% compared to 3.29% of cases. In theory, the ranked sampling criterion is conservative if the assumption holds that documents chosen by machine learning are not less likely to be relevant than those chosen at random. Based on our experiments, this assumption seems reasonable and accounts for the higher recall. Because the ranked quasi-sampling criterion can flexibly choose its sample, whereas the random criterion has to wait for a random sample to be triggered, the criterion is also triggered earlier, as it can make use of more data. This accounts for the higher work savings.

The baseline sampling criteron (Fig. 5c) misses the 95% recall target in 39.67% of cases, while the most common work saving is 0%. This is in line with our expectations that, due to random sampling error, the expected number of documents will often be overestimated or underestimated, resulting in zero work savings or poor recall.

The heuristic stopping criteria, both for 50 consecutive irrelevant results (Fig. 5d—IH50) and for 200 irrelevant results (Fig. 5e), also perform unreliably. Although the mean work saved for IH50 is 41%, the target is missed in 39% of cases. The cases below the horizontal grey line indicate instances where work has been saved at the expense of achieving the recall target.

**Fig. 6:**
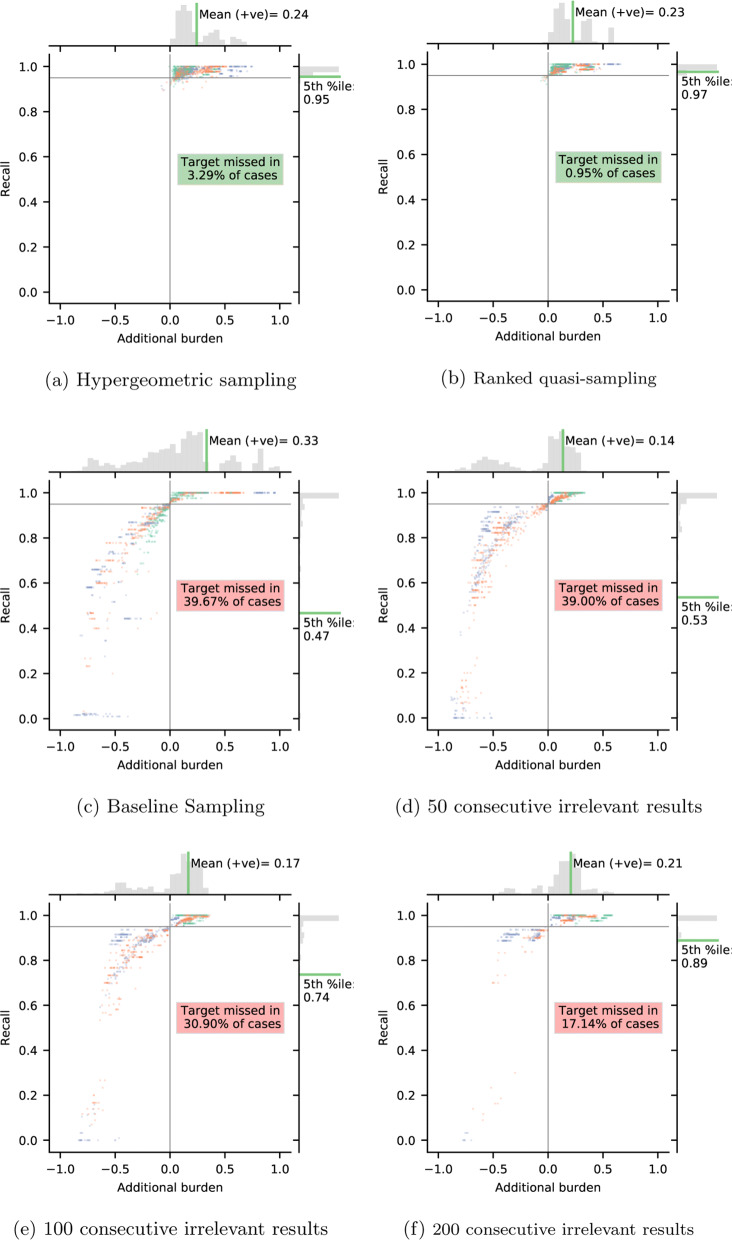
Distribution of recall and additional burden after each stopping criterion. Additional burden is the work saved when the criterion was triggered minus the work saved when the target was reached. Colouring of data points as in Fig. 5

In Fig. 6, we rescale the *x* axis, calling it additional burden, which is simply the work saved when the criterion is triggered minus the work saved when the recall target was actually achieved. This measure indicates whether the stopping criterion was triggered too early (negative values) or too late (positive values). The figure directly highlights the trade-offs involved in deciding when to stop screening: For our criteria, there is mostly a small additional burden which comes with the necessity to make sure the desired recall target has been reached and reject the null hypothesis that this has not been the case. For the other criteria, there are many cases in which additional burden is negative, i.e. the criterion has been triggered too early. In these cases, however, the desired recall is missed.

**Fig. 7:**
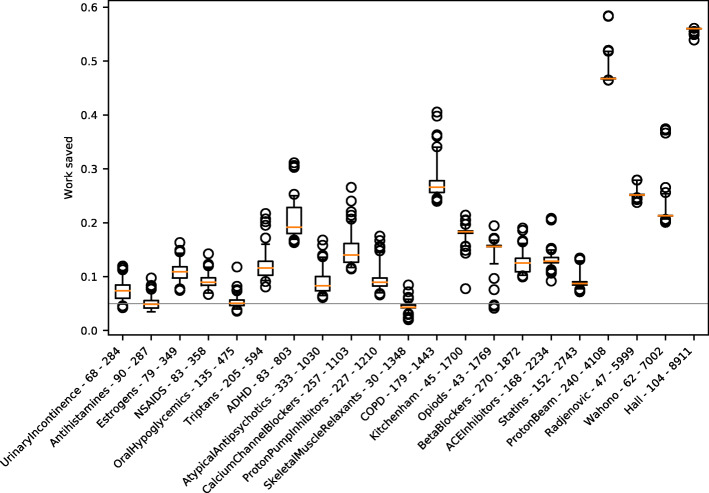
Work saved for the ranked quasi-sampling method in each dataset. Labels show the number of relevant documents and the total number of documents. The datasets are presented in order of the number of documents. The whiskers represent the 5th and 95th percentiles. The grey line shows work savings of 5%

To help explain the different work savings that were observed in our experiments, we show the distribution of work savings from our ranked quasi-sampling criterion for each dataset in Fig. 7. In general, higher work savings are possible when the total number of documents is larger. However, in datasets with a low proportion of relevant documents, many documents need to be screened to achieve a high confidence that there are only few relevant documents remaining in the unseen ones. Therefore, smaller work savings are possible.

**Fig. 8:**
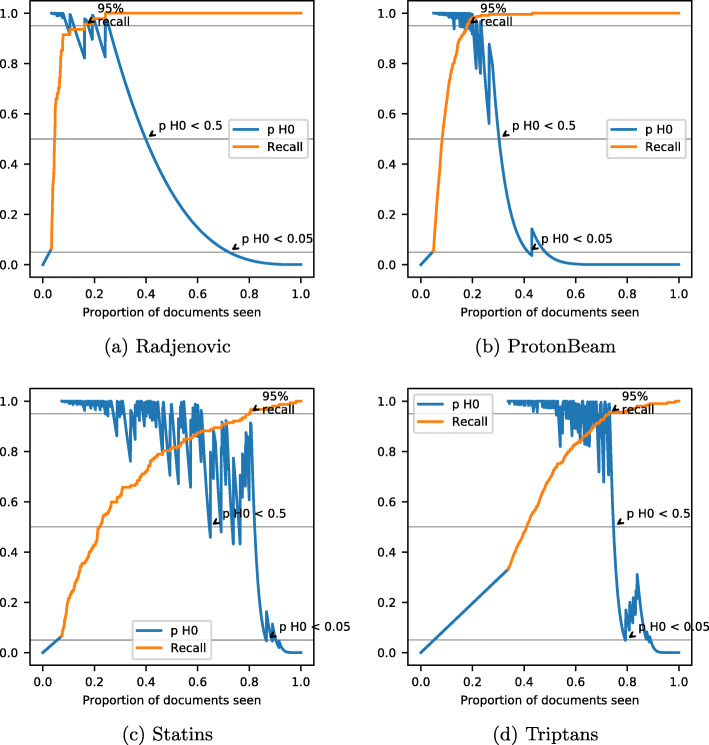
The path of recall (yellow) and the *p* value of H0 for four different datasets

Figure 8 shows the recall and the *p* value for the null hypothesis for the iteration where the recall target is reached first for four datasets. Although the 95% recall target is achieved very quickly in the Radjenovic dataset, the null hypothesis cannot be excluded until much later. This is because the dataset has only 47 relevant documents out of a population of 5999. After the 95% recall target was achieved, 45 out of 47 relevant documents had been seen and 5029 documents remained. The null hypothesis was therefore that 3 or more of these 5029 documents were relevant, which requires a lot of evidence to disprove. The burden of proof was smaller in the case of the Proton Beam dataset: at the point that the 95% recall threshold was reached, the null hypothesis to disprove was that a minimum of 13 out of 3369 remaining documents were relevant.

The Statins and Triptans datasets show how the criterion performs when the machine learning model has performed poorly in predicting relevant results. In each case, 95% recall is achieved with close to 20% of documents remaining. With fewer documents remaining, it takes fewer screening decisions to rule out the possibility that the number of relevant documents left is incompatible with the achievement of the recall target.

## Discussion

Our results show that it is possible to use machine learning to achieve a given level of recall with a given level of confidence. The trade-off for achieving recall reliably is that the work saving achieved is less than the maximum possible work saving. However, for large datasets with a significant proportion of relevant documents, the additional effort required to satisfy the criterion will be small compared to the work saved by using machine learning. This makes the approach well suited to broad topics with lots of literature. In other words, it is precisely where machine learning will be most useful that the additional effort will be small.

Different use cases for machine learning enhanced screening may also carry different requirements for recall, or different tolerances for uncertainty. These can be flexibly accommodated within our stopping criterion. Importantly, the ability to make statements about the authors’ confidence in achieving a given recall target makes it possible to clearly communicate the implications of using machine learning enhanced screening to readers and reviewers who are not machine learning specialists. This is extremely important in live systematic reviews.

Our criteria have the further advantage that they are independent of the choice or performance of the machine learning model. If a model performs badly at discerning relevant from irrelevant results, the only consequence will be that the work saved will be low. With other criteria, this may result in poor recall. When using machine learning for screening, poor recall can result in biassed results, while low work savings represent no loss to the reviewer as compared to not using machine learning.

One caveat in the derivation of our criteria is that we did not address the problem of multiple testing formally. Such a derivation is mathematically challenging and beyond the scope of this paper. However, the performance of the criteria shows that this is of limited practical concern. Formally describing screening procedures with iterative testing should be a next step towards even more rigourous stopping criteria and should be fully worked out in future research.

So far, systematic review standards have no way of accommodating screening with machine learning. We hope that the reliability and clarity of reporting offered by our stopping criteria make them suitable for incorporation into standards, so that machine learning for systematic review screening can fulfil its promise of reducing workload and making more ambitious reviews tractable.

## Conclusion

This paper demonstrates the drawbacks of existing stopping criteria for machine learning approaches to document screening, particularly with regard to reliability. We propose a simple method that delivers reliable recall, independent of machine learning approach or model performance. Our statistical stopping criteria allow users to easily communicate the implications of their use of machine learning, making machine learning enhanced screening ready for live reviews.

## Data Availability

All computational steps required to reproduce this analysis are documented online at https://github.com/mcallaghan/rapid-screening.

## Footnotes

1 Although Shemilt et al. [12] employ a method to choose a sample size based on uncertainty, they fail to acknowledge the potential implications for recall of their choice. Their margin of error of 0.0025 and observed proportion of relevant studies of 0.0005 translate to estimates of 400±451 relevant results. To reduce the margin of error to ±5*%* of estimated relevant studies, they would have had to screen 638,323 out of 804,919 results. See the notebook https://github.com/mcallaghan/rapid-screening/blob/master/analysis/bir_theory.ipynbthat accompanies this paper for a detailed discussion.

2 The notebook, https://github.com/mcallaghan/rapid-screening/blob/master/analysis/hyper_criteria_theory.ipynb, in the github repository accompanying this paper contains a step by step explanation of this method with code and examples.
